# Improved non-uniform subdivision scheme with modified Eigen-polyhedron

**DOI:** 10.1186/s42492-022-00115-2

**Published:** 2022-07-11

**Authors:** Jingjing Zhang, Yufeng Tian, Xin Li

**Affiliations:** 1 School of Mathematical Sciences, Anhui University, Hefei, 230601 Anhui China; 2 School of Mathematical Sciences, University of Science and Technology of China, Hefei, 230026 Anhui China

**Keywords:** Subdivision surface, Eigen polyhedron, Non-uniform Catmull-Clark surface

## Abstract

In this study, a systematic refinement method was developed for non-uniform Catmull-Clark subdivision surfaces to improve the quality of the surface at extraordinary points (EPs). The developed method modifies the eigenpolyhedron by designing the angles between two adjacent edges that contain an EP. Refinement rules are then formulated with the help of the modified eigenpolyhedron. Numerical experiments show that the method significantly improves the performance of the subdivision surface for non-uniform parameterization.

## Introduction

Catmull-Clark surfaces [1] are ubiquitously used in animation owing to their ability to create smooth surfaces with an arbitrary topology. For compatibility with the current standard representation, i.e., non-uniform rational B-spline (NURBS), several subdivision rules are defined to support non-uniform parameterization [2–6]. All methods express knot information by assigning a knot interval to control the mesh edge, and Catmull-Clark surfaces are reproduced if all knot intervals have values of 1. All such subdivision schemes have a vexing problem in that the blending functions for extraordinary points (EPs) can have two local maxima. This problem was solved in ref. [7] using a new technology called an eigenpolyhedron.

**Fig. 1:**
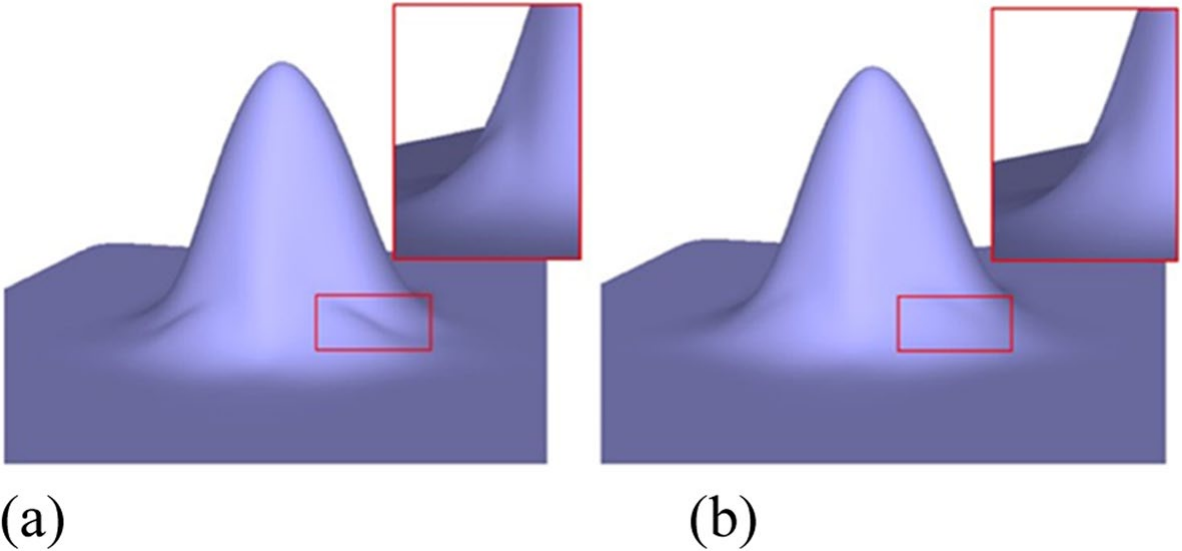
Limit surfaces for a non-uniform EP with a different eigenpolyhedron, using **a** the eigenpolyhedron in ref. [7] and **b** the proposed approach

**Fig. 2:**
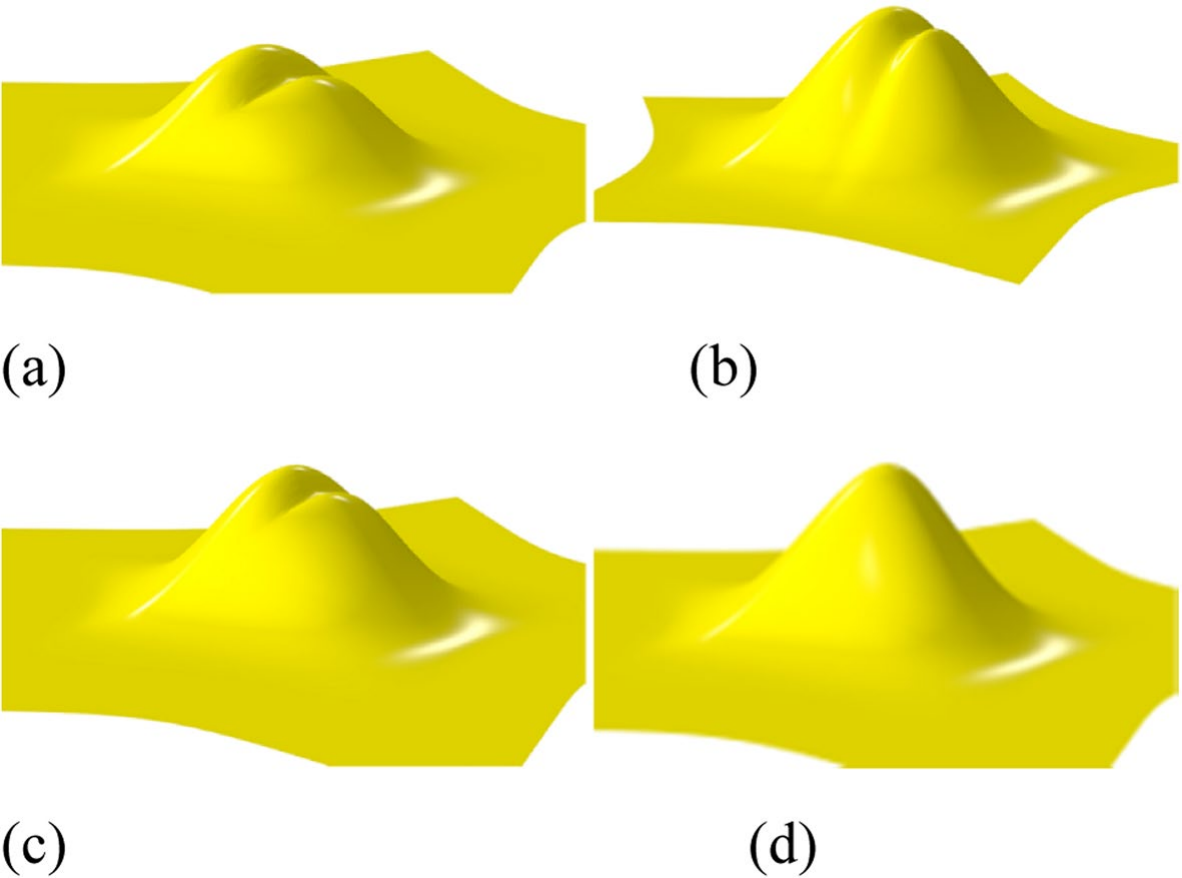
Limit surfaces generalized using **a** NURSS in ref. [2], **b** the scheme in ref. [4], **c** the rule in ref. [6], and **d** the newly developed method

To define the rule provided in ref. [7], the eigenpolyhedron is first defined based on *R*^2^. The final rule is defined under certain constraints when applying the rule on an eigenpolyhedron. The scale and translation of the original polyhedron are obtained and thus different polyhedrons lead to different subdivision rules, which affect the quality of the surface limit. The eigenpolyhedron selected in ref. [7] adopts equal angles between adjacent edges containing an EP. However, in the case of non-uniform knots, owing to the difference in the knot intervals of adjacent edges, the subdivision rules no longer have cyclic symmetry. Thus, making all angles of the eigenpolyhedron equal is not the best choice. Based on this observation, the quality of the subdivision surface was improved in the present study by designing the eigenpolyhedron angles. The numerical results illustrate that the new rules can improve the final limit surface if the ratios of the knot intervals are large. Figure 1 shows a simple example of a valence-5 EP with knot intervals of 1, 15, 1, 1, and 15. Figure 1(a) shows the result of the approach in ref. [7], and Fig. 1(b) shows the result of the newly proposed method. It is clear that the new method can produce a limit surface with higher quality. The limit surfaces produced by the other non-uniform subdivision schemes are shown in Fig. 2. It can be seen that both the present scheme and the rule in ref. [7] can produce much better limit surfaces, detailed comparisons of which can be found in ref. [7]. The following focuses only on comparisons between the present scheme and that in ref. [7].

### Prior work

A subdivision is a useful method for geometric modeling, and is typically generalized from a spline representation to define an arbitrary topology of free-form surfaces. The first two popular types of subdivision are the Doo-Sabin [8] and Catmull-Clark [1] subdivisions, which extend uniform bi-quadratic B-spline and uniform bi-cubic B-spline surfaces to an arbitrary control grid. Subsequently, many subdivision rules have been defined for different control grids and different applications, such as a loop subdivision [9], $\sqrt{3}$-subdivision [10], 4–8 subdivision [11, 12], quad/triangle subdivision [13, 14], four-point interpolatory subdivision [15], and butterfly scheme [16, 17].

NURBS is the dominant standard in industrial design. It is therefore important to construct non-uniform subdivision schemes to apply a subdivision to CAD [18, 19]. For this purpose, Sederberg et al. [2] proposed the first non-uniform B-spline subdivision scheme called NURSSes. In addition, Müller et al. [3] designed a new subdivision surface by forcing the knots of the edge containing the EP to be equal. Cashman et al. [4] proposed a local refinement rule such that the largest knot interval is no more than twice as large as the smallest knot interval at an EP. In ref. [6], a subdivision rule is defined for analysis-suitable T-splines [20] and a new heuristic rule for EPs. All of these subdivision schemes have a vexing problem in that the blending functions for EPs can have two local maxima. The problem was solved in ref. [7] using an eigenpolyhedron. The technology was applied to construct a non-uniform Doo-Sabin subdivision scheme [21] and design a subdivision rule supporting sharp features [22]. Further improvements include a proven *G*^1^ continuous non-uniform subdivision scheme [23] and an optimal convergence rate non-uniform subdivision scheme [24].

### Eigenpolyhedron

This study focuses on the variations in a Catmull-Clark subdivision. For such a subdivision rule, focus was on a valence-n vertex *V*^*k*^ at level *k*. Suppose its neighboring face points are ${F}_i^k$ and neighboring edge points are ${E}_i^k,k=0,1,\dots, n-1$. The subdivision rule computes a set of points *V*^*k* + 1^, ${F}_i^{k+1}$, and ${E}_i^{k+1}$ as a linear combination of *V*^*k*^, ${F}_i^k$, and ${E}_i^k$, respectively. This relation can be written as

1 $${P}^{k+1}={M}^k{P}^k$$

where ${P}^k={\left[{F}_0^k,\dots, {F}_{n-1}^k,{E}_0^k,\dots, {E}_{n-1}^k,{V}^k\right]}^T$, *M*^*k*^ is a (2*n* + 1) × (2*n* + 1) stochastic matrix, and *k* is the refinement level. Matrix *M*^*k*^ is called a subdivision matrix. In this study, it is assumed that *M*^*k*^ is invariant with level *k*, which is denoted as *M* in the following:

The above subdivision relation can be used for any control grid, *P*^*k*^. The eigen-polyhedron concept attempts to study the behavior of the above rule by applying it to a control grid in *R*^2^, which is denoted as ${\hat{P}}^k$ in the following.

**Definition 2.1** A polyhedron ${\hat{P}}^0$ is an eigenpolyhedron of *M* if

2 $${\hat{P}}^1=M{\hat{P}}^0=\lambda {\hat{P}}^0+I{\hat{T}}^0$$

where $$\lambda \in R,{\hat{T}}^0\in {R}^2,$$$${\hat{V}}^0$$ of $${\hat{P}}^0$$ is (0, 0), *M* is a (2*n* + 1) × (2*n* + 1) matrix whose rows sum to 1, and *I* is a (2*n* + 1) × 1 vector whose elements are all 1 s.

It can be seen from the definition that, if ${\hat{P}}^0$ is an eigenpolyhedron of *M*, the following equation is found through induction:

3 $${\hat{P}}^k=M{\hat{P}}^{k-1}={\lambda}^k{\hat{P}}^0+{\sum}_{i=0}^{k-1}{\lambda}^iI{\hat{T}}^0={\lambda}^k{\hat{P}}^0+I{\hat{V}}^k$$

Denoting ${\hat{T}}^k={\hat{V}}^{k+1}-{\hat{V}}^k$, it is easy to obtain ${\hat{T}}^{k+1}=\lambda {\hat{T}}^k={\lambda}^k{\hat{T}}^0,k=1,2\dots$ . Thus, *M* has an eigenpolyhedron if *M* has two identical eigenvalues *λ*, and the corresponding eigenvectors are the two columns of ${\hat{P}}^0-I\frac{{\hat{V}}^1}{1-\lambda }$. Further details are provided in ref. [7].

**Fig. 3:**
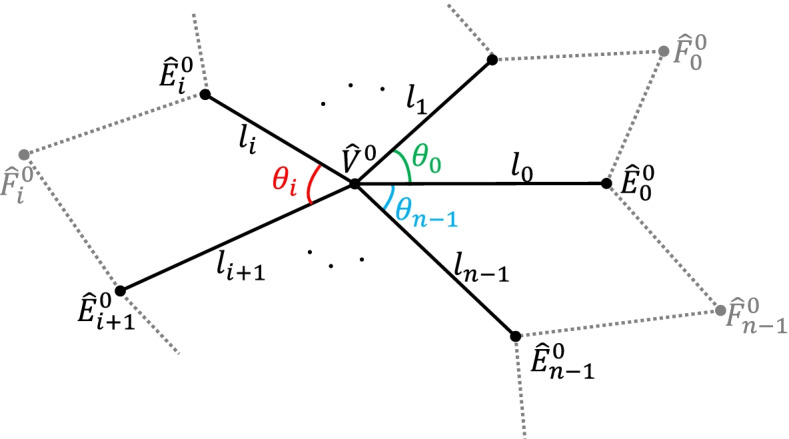
Notations for an eigen-polyhedron ${\hat{P}}^0$ for an EP, where ${\hat{V}}^0$ is (0, 0), *l*_*i*_ are the lengths of edges ${\hat{V}}^0{\hat{E}}_i^0$, and *θ*_*i*_ are the angles between ${\hat{V}}^0{\hat{E}}_i^0$ and ${\hat{V}}^0{\hat{E}}_{i+1}^0$. The edge points ${\hat{E}}_i^0$ in ${\hat{P}}^0$ are determined by *l*_*i*_ and *θ*_*i*_

To define an eigenpolyhedron ${\hat{P}}^0$, it is necessary to define the vertices ${\hat{F}}_i^0$ and ${\hat{E}}_i^0$ of ${\hat{P}}^0,i=0,1,\dots, n-1$. Vertices ${\hat{E}}_i^0$ can be determined based on the lengths *l*_*i*_ of edges ${\hat{V}}^0{\hat{E}}_i^0$ and angles *θ*_*i*_ between ${\hat{V}}^0{\hat{E}}_i^0$ and ${\hat{V}}^0{\hat{E}}_{i+1}^0$, as shown in Fig. 3.

The Catmull-Clark scheme and non-uniform bi-cubic B-spline refinement rule both have a corresponding eigenpolyhedron. For the Catmull-Clark subdivision, the eigenpolyhedron for a valence-n EP can be defined as follows:

4 $${\hat{V}}^0=\left(0,0\right)$$

5 $${\hat{E}}_i^0=\left(\cos \left(\frac{2 i\pi}{n}\right),\sin \left(\frac{2 i\pi}{n}\right)\right)$$

6 $${\hat{F}}_i^0=\upgamma \left({\hat{E}}_i^0+{\hat{E}}_{i+1}^0\right)$$

where $\upgamma =\frac{4}{c_n+1+\sqrt{\left({c}_n+9\right)\left({c}_n+1\right)}},{c}_n=\cos \left(\frac{2\pi }{n}\right)$. The responding translation factor $\lambda =\frac{1+\gamma }{4\gamma }=\frac{c_n+5+\sqrt{\left({c}_n+9\right)\left({c}_n+1\right)}}{16},$ and ${\hat{T}}^0=\left(0,0\right)$.

For a non-uniform bi-cubic B-spline, its eigenpolyhedron can be defined as

7 $${\hat{V}}^0=\left(0,0\right)$$

8 $${\hat{E}}_i^0=\frac{2{d}_i+{d}_{i+2}}{3}\left(\cos \left(\frac{i\pi}{2}\right),\sin \left(\frac{i\pi}{2}\right)\right)$$

9 $${\hat{F}}_i^0={\hat{E}}_i^0+{\hat{E}}_{i+1}^0$$

where *d*_*i*_ is the knot interval, for which *i* = 0, 1, 2, 3. In this case, the corresponding translation factor $\lambda =\frac{1}{2}$, and ${\hat{T}}^0=\left(\frac{d_0-{d}_2}{6},\frac{d_1-{d}_3}{6}\right)$.

It can be seen that all angles are the same in the Catmull-Clark scheme and NURBS eigenpolyhedron. Thus, the angles of the eigenpolyhedron in ref. [7] were set to equal values. However, the experimental results indicate that equal angles for the eigenpolyhedron will lead to an unsatisfactory limit surface if the ratio of the knot intervals is sufficiently large. This motivated us to design non-uniform angles for the eigenpolyhedron, as well as a new non-uniform subdivision scheme, which will be discussed in the next section.

### Organization

The remainder of this paper is organized as follows. Methods section discusses the modification of the eigenpolyhedron for a non-uniform Catmull-Clark subdivision surface. Result section presents examples of the subdivision surfaces and compares the effectiveness of the proposed method with that of previous approaches. In the final two sections, the Conclusion and Discussion are provided.

## Methods

### Modified eigenpolyhedron

This section provides a detailed definition of the modified eigenpolyhedron. To construct a subdivision using eigenpolyhedron-based technology, it is first necessary to design an eigenpolyhedron for an EP, from which a refinement matrix is created. In the following, an eigenpolyhedron is designed for an EP of valence *n*. For the eigenpolyhedron, it is necessary to define vertices ${\hat{E}}_i^0$ and ${\hat{F}}_i^0,i=0,1,\dots, n-1$. Vertices ${\hat{E}}_i^0$ can be computed through the lengths *l*_*i*_ of edges ${\hat{V}}^0{\hat{E}}_i^0$ and angles ${\theta}_i=\angle {\hat{E}}_i^0{\hat{V}}^0{\hat{E}}_{i+1}^0$.

**Fig. 4:**
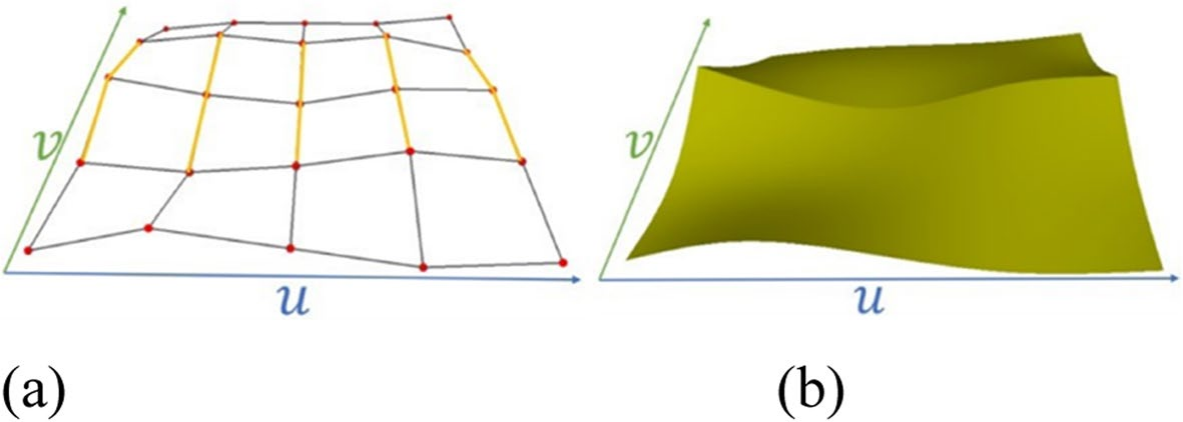
Notations for a crease edge in a NURBS surface, where the edges related to zero knot intervals are shown in yellow. **a** Control mesh; **b** NURBS surface

The definition of the angles was inspired by a study on B-splines. For a bicubic B-spline surface, the zero-knot intervals are equivalent to double knots. The surface patches corresponding to the knot degenerate into B-spline curves, as shown in Fig. 4. Thus, in the case of an EP, if one knot interval is extremely small compared to the other knot intervals, the two adjacent angles should be close to $\frac{\pi }{2}$.

Suppose that the knot intervals of the adjacent edges are denoted by *d*_*i*_, *i* = 0, 1, …, *n* − 1, and that ${k}_i=\frac{n{d}_i{d}_{i+1}}{\sum_{i=0}^{n-1}{d}_i{d}_{i+1}}$, ${\theta}_i^{pre}$ is then defined as

10 $${\theta}_i^{pre}=\left\{\begin{array}{c}\frac{\pi }{2}-\left(2-\frac{8}{n}\arctan \left({k}_i\right)\right),{k}_i<1\\ {}\left(\frac{4}{n}-\frac{1}{2}\right)\pi +\left(2-\frac{8}{n}\right)\arctan \left(\frac{1+{k}_i}{2}\right),{k}_i\ge 1\end{array}\right.$$

The sum of these initial values is denoted by ${\theta}_{sum}^{pre}={\sum}_{i=0}^{n-1}{\theta}_i^{pre}$. If ${\theta}_{sum}^{pre}=2\pi$, let ${\theta}_i={\theta}_i^{pre}$ be the eigenpolyhedron angle. It is obvious that the condition ${\theta}_{sum}^{pre}=2\pi$ can be satisfied when *n* = 4 or *d*_0_ = *d*_1_ = … = *d*_*n* − 1_. However, if *n* ≠ 4 and *d*_*i*_ are not the same, ${\theta}_{sum}^{pre}=2\pi$ cannot generally be obtained. Therefore, it is necessary to modify the values of ${\theta}_i^{pre}$. The maximum value in the set $\left\{{\theta}_i^{pre},i=0,1,\dots, n-1\right\}$ can first be found, and is then denoted by ${\theta}_{max}^{pre}$, whereas the number of maximums in the set is denoted by *N*_*max*_. Next, some initial values ${\theta}_i^{pre}$ are modified in Eq. (10) such that the condition ${\theta}_{sum}^{pre}=2\pi$ can be satisfied.

1. *n* ≠ 3
  If $${\theta}_{max}^{pre}\bullet {N}_{max}<2\pi$$,
    for *i* = 0, 1, …, *n* − 1,
    ➢ if the value of ${\theta}_i^{pre}$ is not equal to that of ${\theta}_{max}^{pre}$, let *θ*_*i*_ be $\frac{2\pi -{\theta}_{max}^{pre}\bullet {N}_{max}}{\theta_{sum}^{pre}-{\theta}_{max}^{pre}\bullet {N}_{max}}\bullet {\theta}_i^{pre}$, and
    ➢ if *θ*_*i*_ is equal to that of ${\theta}_{max}^{pre}$, the values of the other elements in the set remain unchanged.
  If ${\theta}_{max}^{pre}\bullet {N}_{max}\ge 2\pi$ and *N*_*max*_ = *n*,
    for *i* = 0, 1, …, *n* − 1,
    reassign *θ*_*i*_ to $$\frac{2\pi }{\theta_{sum}^{pre}}\bullet {\theta}_i^{pre}$$.
  If ${\theta}_{max}^{pre}\bullet {N}_{max}\ge 2\pi$ and *N*_*max*_ < *n*,
    let *N*_1/2_ = max {*N*_*max*_, *n* − *N*_*max*_}.
    For *i* = 0, 1, …, *n* − 1,
    ➢ if *θ*_*i*_ is equal to that of $${\theta}_{max}^{pre}$$, let *θ*_*i*_ be $$\frac{2\pi \bullet {N}_{1/2}}{n{\theta}_{max}^{pre}\bullet {N}_{max}}$$$$\bullet {\theta}_i^{pre}$$, and
    ➢ if the value of *θ*_*i*_ is not equal to that of $${\theta}_{max}^{pre}$$, let *θ*_*i*_ be $$\frac{2\pi \left(n-{N}_{1/2}\right)}{n\left({\theta}_{sum}^{pre}-{\theta}_{max}^{pre}\bullet {N}_{max}\right)}$$$${\theta}_i^{pre}$$.
2. *n* ≠ 3
  In this case, the sum of all ${\theta}_i^{pre}$ values in Eq. (10) can be computed as no greater than 2*π*. In addition, let ${\theta}_i=\frac{2\pi }{\theta_{sum}^{pre}}\bullet {\theta}_i^{pre},i=0,1,\dots, n-1.$

The lengths *l*_*i*_ of edge ${\hat{V}}^0{\hat{E}}_i^0$ are defined similarly to those in ref. [7], as illustrated in Eq. (11):

11 $${l}_i=\frac{d_i+{d}_i^{-}+{d}_i^{+}}{3}$$

where

$${\displaystyle \begin{array}{c}{d}_i^{+}={\sum}_{j=i,\mid i-j\mid \le \frac{n}{4}}^{i+n-1}{d}_j\cos \left(\frac{2\left(i-j\right)\pi }{n}\right)\\ {}{d}_i^{-}={\sum}_{j=i,\left|i-j\right|>\frac{n}{4}}^{i+n-1}{d}_j\cos \left(\frac{2\left(i-j\right)\pi }{n}\right)\end{array}}$$

The vertices ${\hat{E}}_i^0$ of the eigenpolyhedron ${\hat{P}}^0$ can then be obtained as follows:

12 $$\left\{\begin{array}{c}{\hat{E}}_0^0=\left({l}_0,0\right)\ \\ {}{\hat{E}}_i^0={l}_i\left(\cos \left({\sum}_{j=0}^{i-1}{\theta}_j\right),\sin \left({\sum}_{j=0}^{i-1}{\theta}_j\right)\right),i=1,2,\dots, n-1\end{array}\right.$$

The face points ${\hat{F}}_i^0$ of the eigenpolyhedron ${\hat{P}}^0$ are as follows:

13 $${\hat{F}}_i^0=\upgamma \left({\hat{E}}_i^0+{\hat{E}}_{i+1}^0\right),i=0,1,\dots, n-1$$

The scale *λ* is same as before, i.e.,

14 $$\lambda =\frac{1+\gamma }{4\gamma }=\frac{c_n+5+\sqrt{\left({c}_n+9\right)\left({c}_n+1\right)}}{16}$$

where $$\upgamma =\frac{4}{c_n+1+\sqrt{\left({c}_n+9\right)\left({c}_n+1\right)}},{c}_n=\cos \left(\frac{2\pi }{n}\right)$$.

**Fig. 5:**
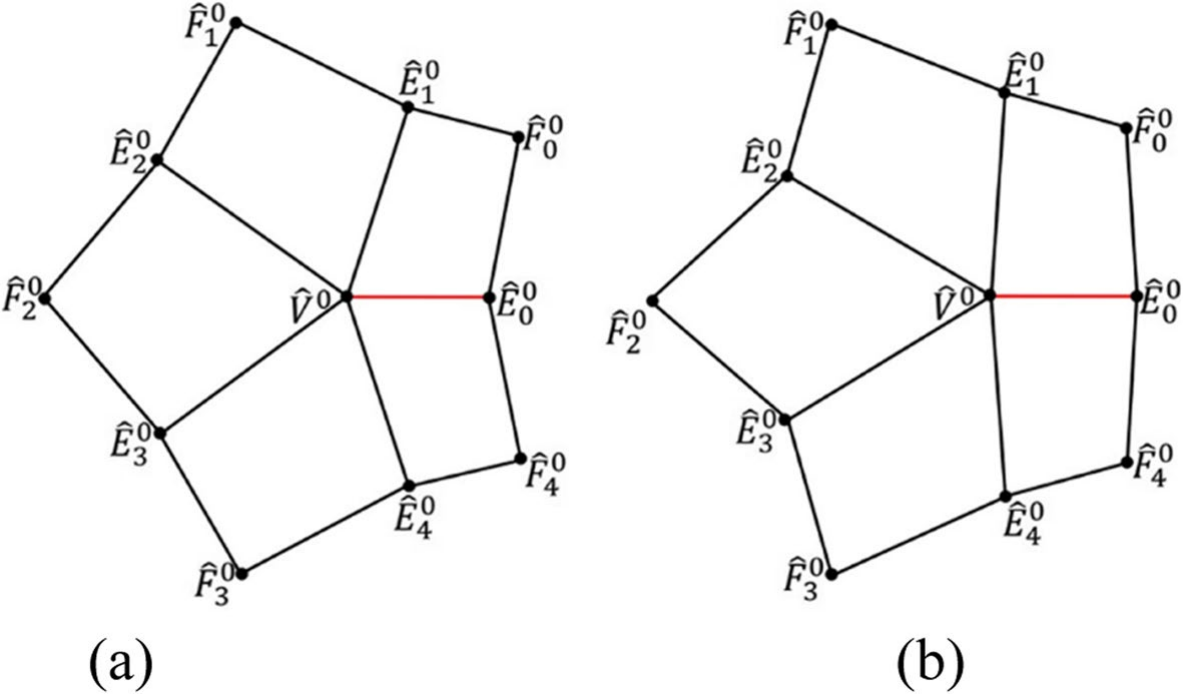
An eigenpolyhedron for a valence-5 EP, where the black edges have knot intervals of 10, and the red edge has knot intervals of 1. **a** Using the approach in ref. [7]; **b** Using the proposed method

Figure 5 shows an example of different eigenpolyhedrons for a valence-5 EP.

### New subdivision rule based on modified eigenpolyhedron

A refinement matrix *M* must satisfy the definition of an eigenpolyhedron. If all knot intervals are equal, *M* must specialize in a Catmull-Clark refinement. If the valence of the point is 4, *M* must specialize in a NURBS refinement. Creating a refinement matrix *M* is equivalent to applying the design vertex, face, and edge-point rules. The other refinement processes are the same as those in ref. [7].

#### Vertex point rule

15 $${\hat{V}}^{k+1}=\frac{n-3}{n}\ {\hat{V}}^k+\frac{3}{n}\frac{\sum_{i=0}^{n-1}\left({m}_i{H}_i^k+{f}_i{G}_i^k\right)}{\sum_{i=0}^{n-1}\left({m}_i+{f}_i\right)}$$

where

$${\displaystyle \begin{array}{l}{H}_i^k={g}_i{\hat{E}}_i^k+\left(1-{g}_i\right){\hat{V}}^k\\ {}{G}_i^k={g}_i\left(1-{g}_{i+1}\right){\hat{E}}_i^k+{g}_{i+1}\left(1-{g}_i\right){\hat{E}}_{i+1}^k+{g}_i{g}_{i+1}{\hat{F}}_i^k+\left(1-{g}_i\right)\left(1-{g}_{i+1}\right){\hat{V}}^k\\ {}{g}_i=\frac{d_{i-2}+{d}_i+{d}_{i+2}}{d_{i-2}+4{d}_i+{d}_{i+2}},{f}_i={\prod}_{j=1,j\ne i,i+1}^n{d}_j^{+},{m}_i={f}_i+{f}_{i-1}\end{array}}$$

Let

16 $${\hat{T}}^0={\hat{V}}^1=\frac{3}{n}\frac{\sum_{i=0}^{n-1}\left({m}_i{H}_i^0+{f}_i{G}_i^0\right)}{\sum_{i=0}^{n-1}\left({m}_i+{f}_i\right)}$$

#### Face point rule

The face point rule is defined with the help of an eigenpolyhedron. According to the definition of an eigenpolyhedron, ${\hat{P}}^1=M{\hat{P}}^0$. Thus,

17 $${\displaystyle \begin{array}{l}{\hat{F}}_i^1={\hat{T}}^0+\lambda {\hat{F}}_i^0\\ {}=\left(1-{\alpha}_{i,1}\right)\left(1-{\alpha}_{i,2}\right){\hat{V}}^0+{\alpha}_{i,1}{\alpha}_{i,2}{\hat{F}}_i^0+{\alpha}_{i,1}\left(1-{\alpha}_{i,2}\right){\hat{E}}_i^0+\left(1-{\alpha}_{i,1}\right){\alpha}_{i,2}{\hat{E}}_{i+1}^0\end{array}}$$

This equation has two functions with two unknowns *α*_*i*, 1_ and *α*_*i*, 2_, which can be solved in the explicit form shown below. Let $${v}_1={\hat{F}}_i^1-{\hat{V}}^0,{v}_2={\hat{F}}_i^1-{\hat{E}}_i^0$$, $${v}_3={\hat{F}}_i^1-{\hat{F}}_i^0,$$$${v}_4={\hat{F}}_i^1-{\hat{E}}_{i+1}^0,$$$${S}_i=\frac{1}{2}{v}_i\times {v}_{i+1},$$$${T}_i=\frac{1}{2}{v}_{i-1}\times {v}_{i+1},$$ and $$D={T}_1^2+{T}_2^2+2{S}_1{S}_3+2{S}_2{S}_4,$$ and thus

$${\alpha}_{i,1}=\frac{2{S}_4}{2{S}_4-{T}_1+{T}_2+\sqrt{D}},{\alpha}_{i,2}=\frac{2{S}_1}{2{S}_1-{T}_1-{T}_2+\sqrt{D}}$$

#### Edge point rule

The edge point rule can be similarly defined. Let

$${\displaystyle \begin{array}{c}{P}_i^1=\left(1-{\alpha}_{i-1,1}\right){\hat{V}}^0+{\alpha}_{i-1,1}{\hat{E}}_{i-1}^0\\ {}{P}_i^2=\left(1-{\alpha}_{i,2}\right){\hat{V}}^0+{\alpha}_{i,2}{\hat{E}}_{i+1}^0\\ {}\begin{array}{c}{P}_i^3=\left(1-{\alpha}_{i-1,1}\right){\hat{E}}_i^0+{\alpha}_{i-1,1}{\hat{F}}_{i-1}^0\\ {}{P}_i^3=\left(1-{\alpha}_{i,2}\right){\hat{E}}_i^0+{\alpha}_{i,2}{\hat{F}}_i^0\end{array}\end{array}}$$

as

18 $${\displaystyle \begin{array}{l}{\hat{E}}_i^1={\hat{T}}^0+\lambda {\hat{E}}_i^0\\ {}=\left(1-{\beta}_{i,1}\right)\left(1-{\beta}_{i,2}\right)\frac{P_i^1+{\hat{V}}^0}{2}+{\beta}_{i,1}{\beta}_{i,2}\frac{P_i^4+{\hat{E}}_i^0}{2}\\ {}+{\beta}_{i,2}\left(1-{\beta}_{i,1}\right)\frac{P_i^3+{\hat{E}}_i^0}{2}+\left(1-{\beta}_{i,2}\right){\beta}_{i,1}\frac{P_i^2+{\hat{V}}^0}{2}\end{array}}$$

The above equation contains two functions with two unknown coefficients, *β*_*i*, 1_ and *β*_*i*, 2_, which can be solved using the same method.

Summarizing the above, the following steps constituting Algorithm 1 are used to obtain a new and improved non-uniform Catmull-Clark surface.

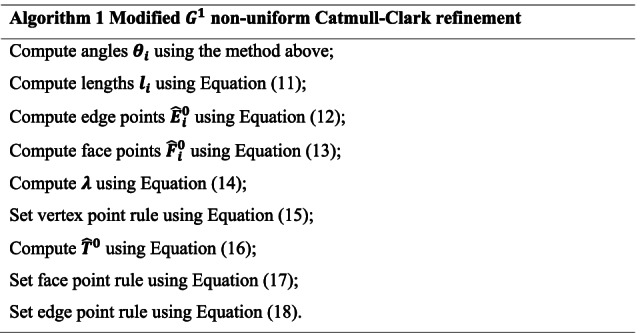



## Results

This section presents some results of the improved subdivision surface using Algorithm 1, and then illustrates the effectiveness of the proposed method. Compared with the method in ref. [7], the refinement in Algorithm 1 recalculates the angles of the eigenpolyhedron, and the other processes are exactly the same as those in ref. [7]. However, the numerical results show that the newly developed algorithm can improve the quality of limit surfaces.

**Fig. 6:**
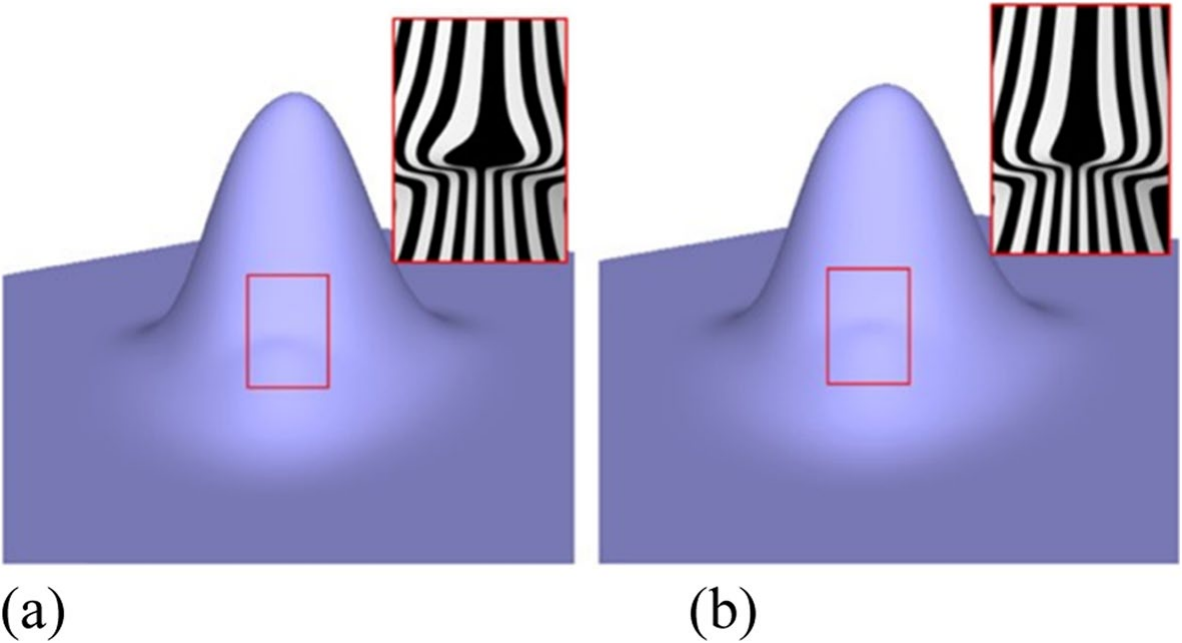
Valence 3, with knot intervals of 1, 10, and 10. **a** Using the approach in ref. [7]; **b** Using the proposed method

**Fig. 7:**
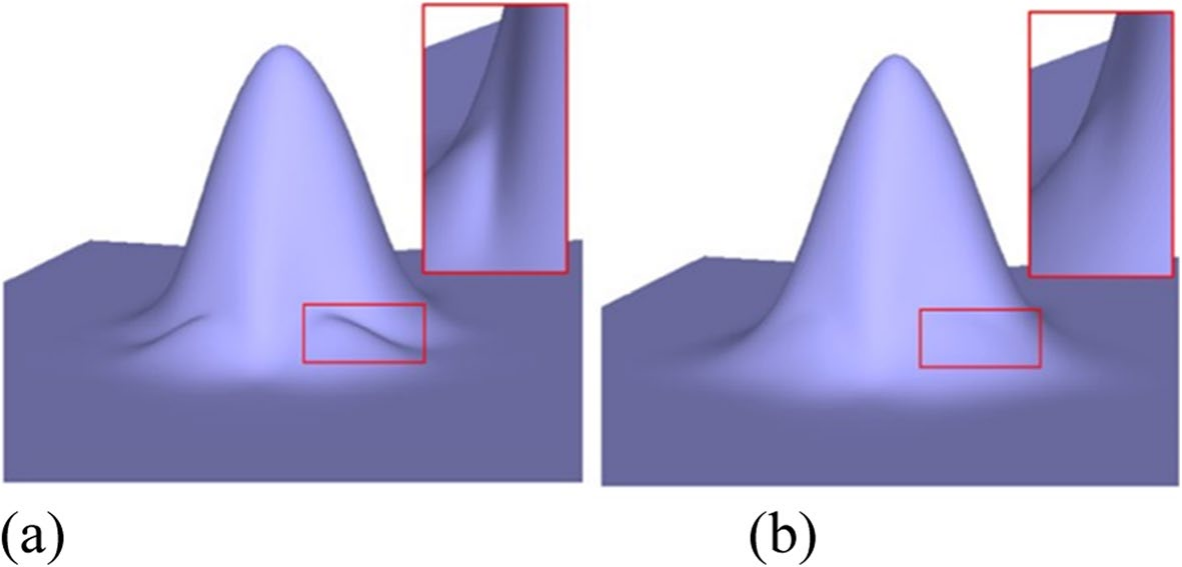
Valence 5, with knot intervals of 1, 1, 10,000, 10,000, and 10,000. **a** Using the approach in ref. [7]; **b** Using the proposed method

**Fig. 8:**
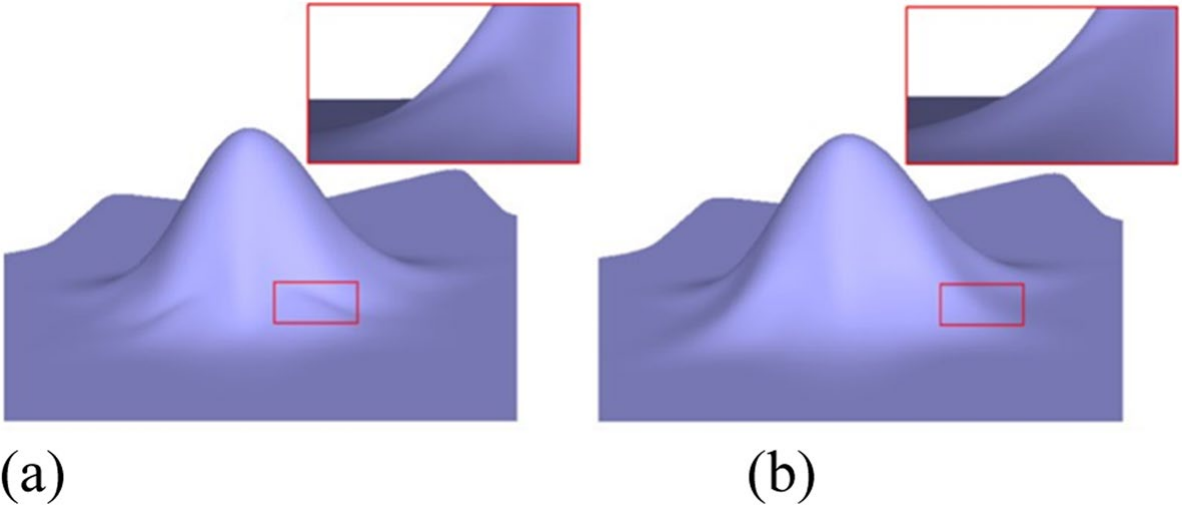
Valence 7, with knot intervals of 100, 100, 1, 1, 100, 100, and 100. **a** Using the approach in ref. [7]; **b** Using the proposed method

**Fig. 9:**
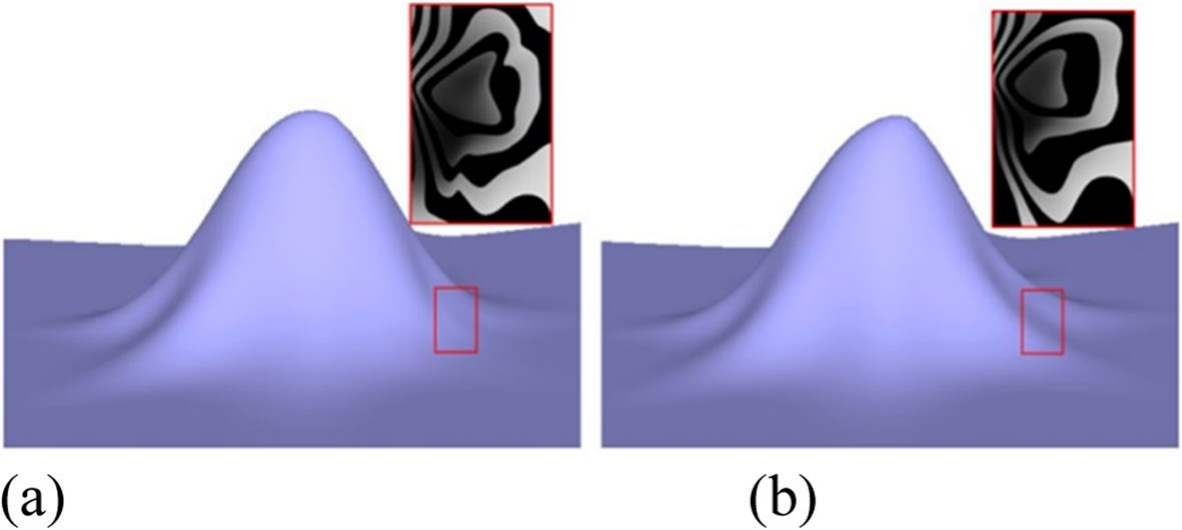
Valence 8, with knot intervals of 100, 100, 1, 1, 100, 100, 1, and 1. **a** Using the approach in ref. [7]; **b** Using the proposed method

**Fig. 10:**
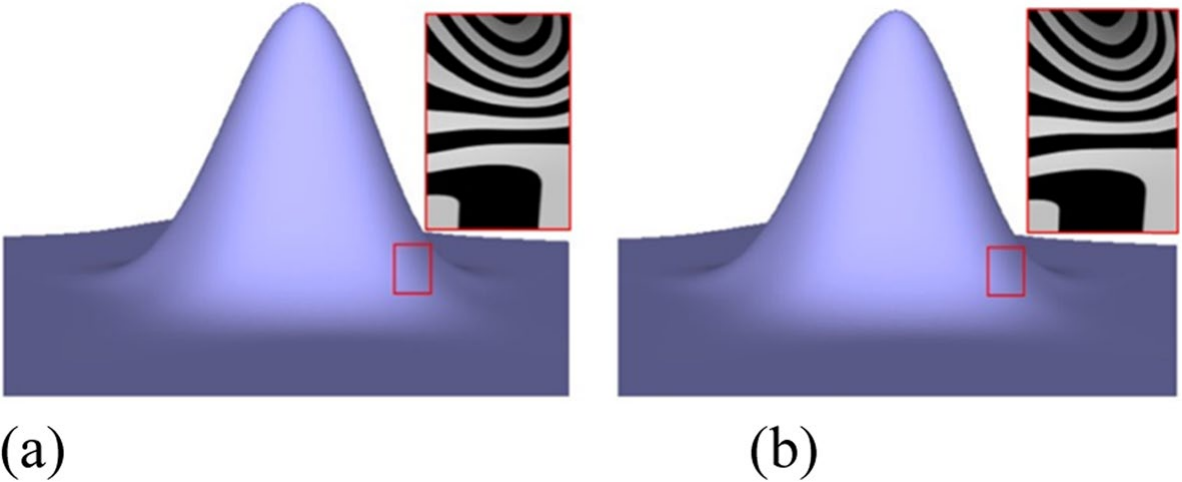
Valence 5, with knot intervals of 1, 1, 1, 5, and 5. **a** Using the approach in ref. [7]; **b** Using the proposed method

Figures 1, 6, 7, 8, 9 and 10 shows examples of blending functions with EPs of valence-3, 5, 7, and 8. The subdivision surfaces prior to the improvement, as shown in these figures, were obtained using the method in ref. [7]. In addition, the improved surfaces are obtained after modifying the angles of the eigenpolyhedron according to the proposed approach. However, a large number of experiments have shown that the eigenpolyhedron technology can eliminate the poor performance of the two local maxima. When the difference between knot intervals is greater, if an equal-angle formula is used for the eigenpolyhedron design in ref. [7], the derivatives of the blending functions do not monotonically decrease within a neighborhood of the EP in the initial control mesh. The comparison results also show that the proposed method is more effective in improving the surface quality if the difference between knot intervals of the adjacent edges to the EP is larger. The differences in knot interval ratios are 100-times greater in Figs. 8 and 9. These results show that the improved method provides a surface as good as that in a uniform case. In addition, a situation in which there is a significant difference in the knot interval ratios was also tested. Figure 7 shows a blending function of valence-5 EP with knot intervals of 1, 1, 10,000, 10,000, and 10,000. Compared with Fig. 1, it can be seen that when the knot interval ratios are extremely large, the eigenpolyhedron design using an equal-angle formula makes the resulting surface behave quite clearly in the above problem, whereas the proposed eigenpolyhedron can solve this problem well.

When there is little difference between the knot intervals, the curved surface obtained using the original equiangular eigenpolyhedron does not demonstrate any problems in the above comparison. The basis functions were tested at EPs of different valences, the results of which showed that the proposed method can retain this advantage. Examples of a valence-5 EP with knot intervals of 1, 1, 1, 5, and 5 are shown in Fig. 10.

**Fig. 11:**
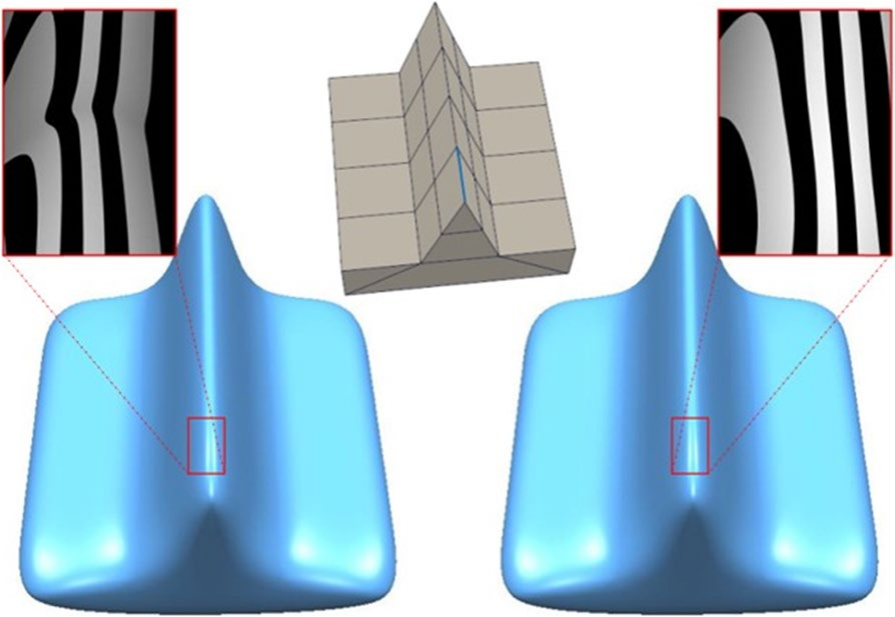
Wedge model

In geometric modeling, the application of the improved eigenpolyhedron method leads to improved models. Figure 11 shows a simple wedge model. The improvement in the surface quality is mainly reflected in the EP of valence-3 within the mesh. The knot intervals corresponding to one of the adjacent edges differ significantly from the knot intervals of the other edges in the mesh. The knot interval of the blue edge is 1, whereas that of the others is 20. It can be seen that the smoothing quality of the resulting surface before the improvement of the eigenpolyhedron is insufficiently optimistic, whereas the quality of the improved surface is satisfactory.

**Fig. 12:**
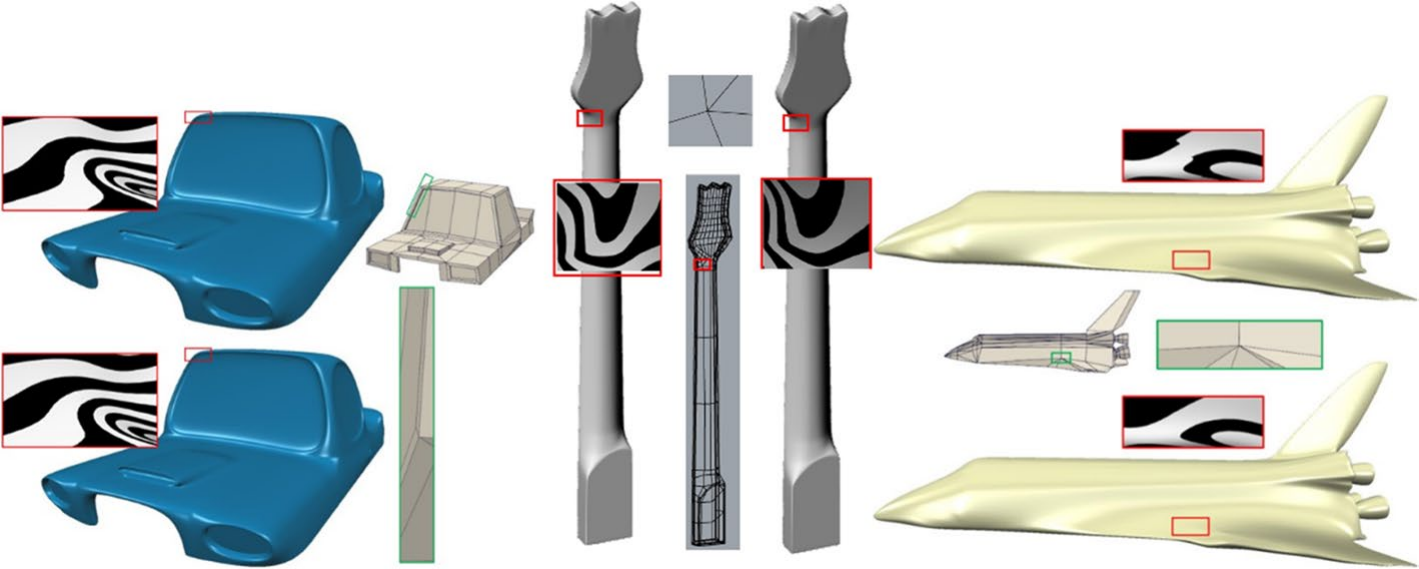
Three real models

Applying the proposed method to more complex industrial geometric modeling can also improve the quality of the subdivision surfaces. The left and middle images of Fig. 12 illustrate a car roof and guitar model, respectively, with an EP of valence-5 for each of the two models. The knot intervals in the mesh of the car roof model were assigned values according to the centripetal parameterization [25]. In the figure, there is a large difference between the knot intervals of the adjacent edges of the EP in each part of the car roof and guitar models. Although the surfaces obtained using the method in ref. [7] and the proposed approach are both *G*^1^, the improved surfaces of the latter are smoother. A complex model was also tested in which the knot intervals of the adjacent edges of the EP were significantly different, as shown on the right side of Fig. 12. In the selected space shuttle model part, there is an EP of valence-7, and the knot intervals are 5000, 1, 1, 5000, 5000, 5000, and 5000. It can be seen that the surface quality is obviously better than that prior to the improvement.

## Conclusions

In this paper, it was specifically shown that different eigenpolyhedron designs from ref. [7] can improve the quality of subdivision surfaces. In addition, a systematic method was developed for designing the angles of the eigenpolyhedron. The effectiveness of this method was verified through numerical experiments. In particular, when the distance between adjacent edges at the EP is large, the proposed method significantly improves the surface quality.

## Discussion

In the present study, only those angles in which the other construction is similar to that in ref. [7] were modified. Thus, the current implementation can be applied within the same framework as that used in ref. [7]. However, the same problems as those in ref. [7] occurred in the present study, the main one being that an analytical proof for a single local maximum and continuous *G*^1^ was unavailable. Thus, similar numerical experiments were conducted to verify these two statements. To verify the existence of a single local maximum, five levels of refinement were applied for each test case, and it was confirmed that the resulting control mesh had a single vertex whose z-coordinate was larger than that of all of the neighbors. An EP is tangent-continuous if the characteristic ring is regular and injective. The regularity and injectivity were verified by subdividing the control mesh of the characteristic map several times and conducting numerical tests to confirm that the determinant of the Jacobian matrix did not change its sign and that no nonlocal intersections occurred. A million different EPs were tested using randomly generated knot intervals of [10^−6^, 1] and valences of *n* = 3, 5, 6, 7, and 8, and it was found that, in every case, the blending function had a single local maximum, i.e., *G*^1^.

In this paper, a surface quality improvement studied based only on the angle configuration factor was described. Improving the surface quality to achieve a class A surface requires further consideration. However, based on this study, a closed-form equation for the limit point of an EP, as well as the tangent vectors for the spoke curves, can be developed in future research, which will be helpful in developing an improved patching solution for non-uniform Catmull-Clark surfaces.

## Data Availability

Not applicable.

## Abbreviations

EP: Extraordinary point
NURBS: Non-uniform rational B-spline
